# An Insight into Pasting and Rheological Behavior of Potato Starch Pastes and Gels with Whole and Ground Chia Seeds

**DOI:** 10.3390/gels8090598

**Published:** 2022-09-18

**Authors:** Greta Adamczyk, Magdalena Krystyjan, Piotr Kuźniar, Przemysław Łukasz Kowalczewski, Inna Bobel

**Affiliations:** 1 Department of Food Technology and Human Nutrition, Institute of Food Technology and Nutrition, University of Rzeszow, 4 Zelwerowicza St., 35-601 Rzeszow, Poland or; 2 Department of Carbohydrates Technology and Cereal Processing, Faculty of Food Technology, University of Agriculture in Krakow, Al. Mickiewicza 21, 31-120 Krakow, Poland; 3 Department of Food and Agriculture Production Engineering, Institute of Agricultural Sciences, Environment Management and Protection, University of Rzeszow, 4 Zelwerowicza St., 35-601 Rzeszow, Poland; 4 Department of Food Technology of Plant Origin, Faculty of Food Science and Nutrition, Poznań University of Life Sciences, 31 Wojska Polskiego St., 60-624 Poznań, Poland; 5 Department of Bakery and Confectionary Goods Technologies, Educational and Scientific Institute of Food Technology, National University of Food Technologies, 68 Volodymyrska St., 01601 Kyiv, Ukraine

**Keywords:** potato starch, chia seeds, pasting characteristic, flow properties

## Abstract

With regard to technological innovations, we applied chia (oilseeds) as a stabilizer additive in a normal and waxy potato starch sample to obtain stable starch-based gels during 20 days of storage. The aim of this study was to investigate the 5% *w*/*w* normal and waxy potato starch pastes (hot samples) and gels (cold samples) with the addition of 1% *w*/*w* whole and ground chia seeds properties as pasting and flow properties of pastes and textural properties of gels. The pasting process using a viscograph showed that normal and waxy potato starch with the addition of chia had a different pasting characteristic. The addition of chia seeds had a greater effect on the properties of normal potato starch than waxy potato starch. From a rheological point of view, starch pastes without chia were less theologically stable as they showed bigger areas of hysteresis loops. Minor changes in the hardness of gels were obtained in normal starch gels with chia seeds during 20 days of storing compared to the samples without chia seeds, whereas in the waxy starch gels, the effect was the opposite.

## 1. Introduction

Starch is a carbohydrate of plant origin found in grains, roots, tubers, and plant fruits. It consists of two factions that differ in structure, called amylose and amylopectin. Amylose molecules are long, linear chains of anhydroglucose rings linked together exclusively by α-1,4-glycosidic bonds [1]. Linear polyglucan chains built like an amylose molecule, but much shorter than it, and additionally connected with α-1,6-glycosidic bonds, form a highly branched structure of amylopectin. The share of amylose fraction in starch depends on its botanical origin and is the main determinant of its functionality, including, first of all, rheological properties [2]. In normal potato starch, the amylose fraction is 16–24%, although, in high amylose starches, it can be even 70% or more, and in the waxy form (high amylopectin) of starch, amylose is present in trace content [3]. Waxy starches, in comparison to the normal form of starch, consist exclusively of amylopectin, and consequently, they have many features that are useful in food processing [4]. On the other hand, normal starch has limited use in the food industry due to its low solubility, strong hydrophilic properties, and unfavorable mechanical properties [3,4].

Starch is an inseparable component of many food products. The most important reason for adding starch is to give the products the right rheological properties. Starch plays a thickening role due to the ability to form gels and gelatinization ability. It affects the structure of products and improves their stability. It is used as an emulsifier, including in sauces, mayonnaises, soups, or pudding masses, and it is as an ingredient in puddings, jellies, ice cream, cake mixes, as well as in breadcrumbs [5]. Moreover, as one of the hydrocolloids, starch is used in the meat industry, e.g., as a partial fat replacement or fat substitute to reduce the caloric content of products [6]. Processing conditions and the presence of various additives in starch gels affect differences in their properties [7].

Currently, the number of people who pay attention to the composition of food products is gradually increasing. Consumers avoid synthetic food additives [8]. Natural or synthetic food additives are added to fulfill specific technological and sensory functions in food products [9]. It becomes desirable to use natural ingredients known to consumers that can be found in domestic kitchens [8,10]. Such ingredients are, for example, chia seeds, which are available on the market and used by consumers for their beneficial effects on health [11].

The Chia plant (*Salvia hispanica* L.) belongs to the *Labiatae* family [12]. Chia has been used in flavoring and folk medicine worldwide as whole seeds, flour, mucilage, and oil. Chia seeds have been interesting in recent years because they were recognized as valuable food raw-material in food technology and human nutrition. The increasing popularity of *Salvia* is due to its nutritional properties thanks to a significant amount of dietary fiber, lipid, protein, and polyphenols [13]. The chemical composition of seeds depends on genetic factors and the influence of the ecosystem in which the plant grew, and contain a high amount of fat (18–30 g/100 g), protein (25–41 g/100 g), carbohydrates (∼42%, of which 34% is dietary fiber and ∼6% is the soluble fiber fraction) and 4–6% ash [14,15,16,17]; EC 2009. Mainly, chia seeds polysaccharide is a tetrasaccharide that is made up of β-D-xylopyranosyl, α-D- glucopyranosyl, and 4-*O*-methyl-α- D-glucopyranosyluronic acid [18]. According to Goh et al. (2016) [19] monosaccharides composition of chia seeds has approximately 35% (*w*/*w*) soluble fraction (consisting of glucose, xylose, galactose, man-nose, and arabinose) and 65% (*w*/*w*) insoluble fraction (consisting of glucose, xylose, and arabinose, which could be derived from the cellulose and hemicellulose, moreover, are in this fraction galactose and mannose). A soluble fraction is a neutral polysaccharide, and an insoluble fraction contains a negatively charged polymer fraction.

When soaked in water, chia seeds form a transparent mucilage that is difficult to separate from seeds, consisting primarily of soluble fiber and mucus. The resulting mucilage has thickening and gelling properties. From a nutritional point of view, the mucilage obtained from hydrated chia seeds contributes to the stabilization of digestive systems comparable to conventional stabilizers, thanks to their water retention and absorption capacity [11]. In the technological aspect, due to water-retaining capacity and the high content of dietary fiber, chia seeds show a good effect on prolonging the freshness, for example, flour products such as bread or pastries [20,21]. In addition, increasing consumer demands are forcing food manufacturers to look for new and unique natural ingredients that can be used in products. Such ingredients include polysaccharides, which can be found in chia mucilage. The usage of chia seeds as a novel food ingredient was authorized by regulation in 2009 [17]. According to the present direction of bakery and pastry products, the addition level was allowed up to 10% chia seeds, and the daily intake should not exceed 15 g [22].

Due to certain disadvantages of starch, such as its thermal instability and tendency to retrograde, its use in the food industry is limited. Therefore, to expand its application, it is necessary to modify starch before its final use as a food ingredient. To extend the range of applicability of starches in the food industry, modifications such as chemical, physical and enzymatic are used [23,24]. However, modified starches do not always meet consumers’ acceptance [24]. In the last few years, many reports have investigated the use of non-starchy polysaccharide hydrocolloids (NPH) to control the course of the pasting and viscosity of corresponding pastes and gels and also improve the functional properties of starch [25,26]. The water holding capacity of chia seed gum is comparable with some plant gums (hydrocolloids), for example, that of guar gum and arabic gum (nearly four times higher than that) [27]. The novel seed gum has received very little attention as a stabilizer in starch gels, despite its popularity for health benefits [28].

Understanding the properties of polysaccharide gel with chia seeds (whole and ground) is important from a technological point of view because such systems are used as stabilizers, thickeners or fat substitutes in food production. [19]. Thus, the aim of our work was to show potential manufacturers that the small number of chia seeds (1% *w*/*w*) used in the normal and waxy potato starch gels formulation may have a positive effect on the technological parameters of gel-based products. Secondly, we wanted to show the differences in starch-based gel parameters in the case of adding chia seeds in whole or ground form to the normal and waxy potato starch.

## 2. Results and Discussion

The analyzed chia seeds were rich in fat and contained up to 29.86%, which corresponds to the range of literature data presented by the authors in their works [29,30,31,32,33]. Fatty acids contained in chia seeds can constitute up to 35%, of which 59.63–59.89% are α-linolenic acid (*ω*3), 20.18–20.56% linoleic acid (*ω*6), and 10.36–10.70% oleic acid (*ω*9) [34,35]. Chia seeds are a good source of plant protein, which accounts was 18.03%, and within the range reported in the literature [17,36,37]. This amount was greater than the protein content in all other cereals (corn–9.4%, rice–6.5%, and wheat–12.6%) [38,39,40]. Therefore, chia seeds can potentially prevent protein-energy malnutrition [41].

The dietary fiber content in 100 g of the tested chia seeds was 36.24%. The amount of this ingredient can vary considerably from 23 to 41% [16,42] (of which about 85–93% are insoluble fractions, and about 7–15% are soluble fractions [16,43]. The chemical composition of chia seeds depends mainly upon environmental and agronomical factors [38,44,45].

**Figure 1:**
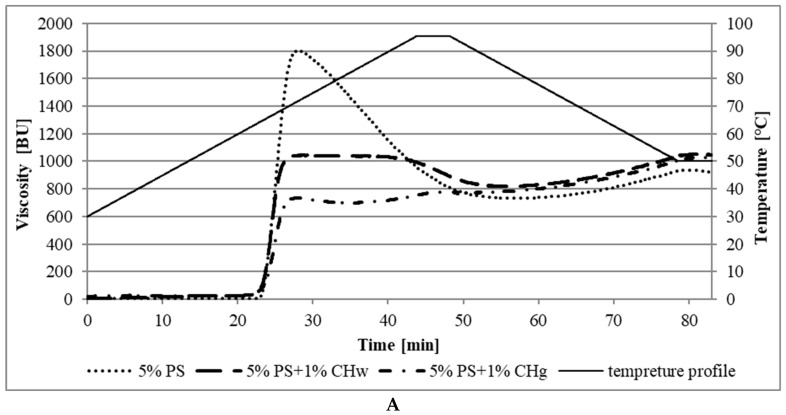

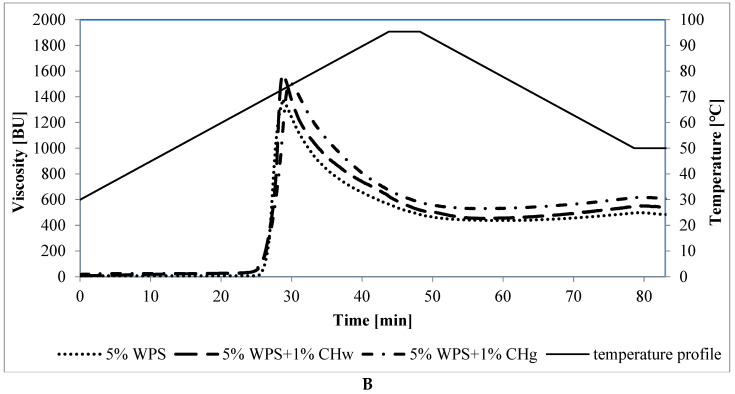
(**A**) Pasting characteristics of 5% PS and its mixtures with 1% of CH (whole and ground seeds) and (**B**) 5% WPS and its mixtures with 1% of CH (whole and ground seeds).

Table 1 and Table 2 present the gelatinization parameters of normal potato starch (5%PS) and waxy potato starch (5%WPS) and their mixtures with whole and ground chia seeds (5%PS + 1%CH_w_, 5%WPS + 1%CH_w_, 5%PS + 1%CH_g_, 5%WPS + 1%CH_g_). The determinations were made using a Brabender viscograph, and their pasting characteristics are presented in Figure 1A,B.

The examined paste of normal starch without the addition of chia seeds showed the values of the initial gelatinization temperature corresponding to the data reported in the literature (64–68 °C) [46,47]. After adding whole chia seeds to PS, the gelatinization temperature dropped significantly to 61.1 °C, and the addition of ground chia seeds also affected the gelatinization temperature of the polysaccharide (62.1 °C). This behavior is due to the fact that chia seeds exhibit a high tendency to absorb water as their structure has numerous free hydroxyl groups that can form bonds with water molecules [48,49]. According to Muñoz et al. (2012) [50], chia seeds can absorb water in amounts 12-fold greater than their own mass.

As seen in Figure 1 and Table 1, PS had the highest maximum viscosity (ƞ_max_) (1802 B.U.) of all tested samples. After adding whole and ground chia seeds to the native starch, the tested mixtures achieved lower values of ƞ_max_, proving that the starch granules’ gelatinization was limited. The gelatinization characteristics, in this case, did not have a clear maximum, and a considerable flattening of the peaks (ƞ_max_) was observed both in the 5%PS + 1%CH_w_ and 5%PS + 1%CH_g_sample as well.

The reduction in the maximum viscosity of the starch solution during pasting is likely the result of competition between starch and chia seeds for water molecules. The insufficient amount of water needed for starch pasting results in strong competition between hydrophilic polymers for water molecules. As a result, a reduction in the viscosity of the overall system is noted. According to Goh et al. (2016) [19], chia seed polysaccharide is not completely soluble, and the rheological properties are reported based on the whole polysaccharide fraction (including soluble and insoluble particles) measured in its entirety. The greater effect of ground seeds on the viscosity of the system is due to the greater accessibility to the water of the soluble fraction and, therefore, stronger competition between the polymers. Soluble dietary fiber absorbs water, turning it into gel-like, highly hydrated masses [11]. Another equally important factor limiting starch pasting might be is the fat in chia seeds. As previous analyses have shown, it accounts for as much as 29.86% of the grain. Literature data also confirm that fat is a factor that strongly limits starch pasting [51].

From the obtained data (Table 2), it can be seen that adding whole chia seeds to WPS, the same as to PS, decreased the parameter T_0_ from 67.6 to 62.2 °C for 5%WPS + 1%CH_w_ and to 64.1°C for 5%WPS + 1%CH_g_. On the other hand, in the studied waxy starch pastes, the addition of whole and ground chia seeds showed a higher ƞ_max_ (1576.0 B.U. and 1509.0 B.U.) versus a waxy starch without the addition of chia seeds (1386.5 B.U). Thus, the relationship was opposite to that of the native starch described above (Table 1).

Normal potato starch was characterized by a higher value of maximum viscosity (1802.0 B.U.) compared to waxy starch (1386.5 B.U.) (Table 1 and Table 2). McPherson and Jane (1999) [52] also observed this phenomenon in their research. This difference may be due to the various proportions of amylose to amylopectin between the starches. Amylose, in combination with amylopectin, allows the starch granules to swell to a greater extent and thus achieve a higher maximum viscosity of the paste. The amylose-free waxy starch grains disperse quickly, resulting in a significantly lower maximum gelatinization viscosity. The fat content of chia seeds also influenced the gelatinization characteristics of the studied starches. The addition of chia seeds to the normal starch reduced the degree of swelling and thus the reduction of ƞ_max_ pastes. In turn, in the case of waxy starch, adding chia increased the paste’s maximum viscosity. According to the literature, the presence of fat in starch pastes largely affects the properties of starch. Fat-forming inclusion complexes with amylose reduce the swelling capacity and the solubility of starch in water and hinder the formation of starch gel [53,54]. Holding of starch pastes at 95 °C resulted in a decrease in the viscosity of the systems, the greatest in the case of starch without the addition of chia seeds. The obtained values of the minimum viscosity (ƞ_min_) of native starch pastes (Table 1) did not differ statistically, and their value for natural starch without the addition of chia seeds was 733.0 B.U., with the addition of whole chia seeds 816.0 B.U. and ground seeds 748.0 B.U. It was similar to waxy starch. The ƞ_min_ of paste without the addition of seeds was 436 B.U., with the addition of whole chia seeds 452 B.U. and ground 528 B.U. (Table 2).

As the systems cool down, the viscosity started to increase again. A more pronounced increase in viscosity was noted in the case of the 5%PS paste (Table 1 and Table 2).

The breakdown parameter (BD) describes the difference between the maximum and minimum viscosity of starch pastes during their heating at the maximum applied temperature (95 °C). In the paste with normal starch, the greatest decrease in viscosity was recorded in the samples without the addition of chia seeds (5%PS), and the value was 1069.0 B.U. The addition of chia seeds reduced the BD to 230.0 B.U. for 5%PS+ 1%CH_w_ and 70 B.U for sample 5%PS+ 1%CH_g_. In the case of waxy starch pastes, the addition of chia seeds (1%CH_w_ and 1%CH_g_) increased the BD values. In the 5%WPS without the addition of chia seeds, the value was 950.5 B.U. with the addition of whole chia seeds 1124.0 B.U. and ground 980.5 B.U. As reported in the literature, a greater decrease in viscosity during heating shows starches with a tendency to less stability of the final product [55,56]. According to Thirathumthavorn and Charoenrein (2006) [57], the greater drop in viscosity with heating causes greater disruption of the starch granules. Based on the bibliography data, it can be concluded that the addition of chia seeds to normal starch pastes increased their stability.

The highest viscosity (1039 B.U.) after cooling to 50 °C (η_50°C_) was recorded in a sample of normal starch with the addition of ground chia seeds (5%PS + 1%CH_g_). The remaining values were 1037 B.U. in 5%PS + 1%CH_w_ and 927.5 B.U. in the 5%PS sample (Table 1). On the other hand, the parameter η_50°C_ in waxy starch had lower values than in the case of normal starch. In the WPS, this value was 483 B.U., in the 5%WPS + 1%CH_w_ 539.5 B.U. and in 5%PS + 1%CH_g_ 607.5 B.U. (Table 2).

**Figure 2:**
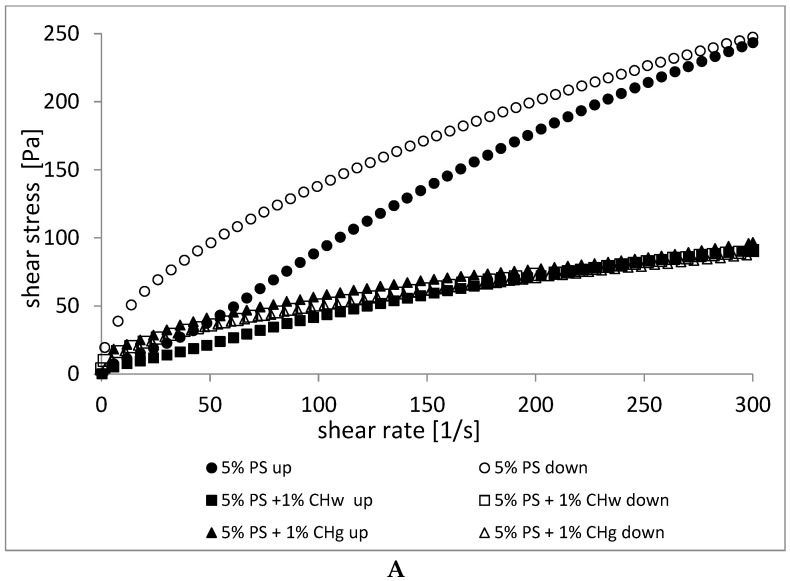

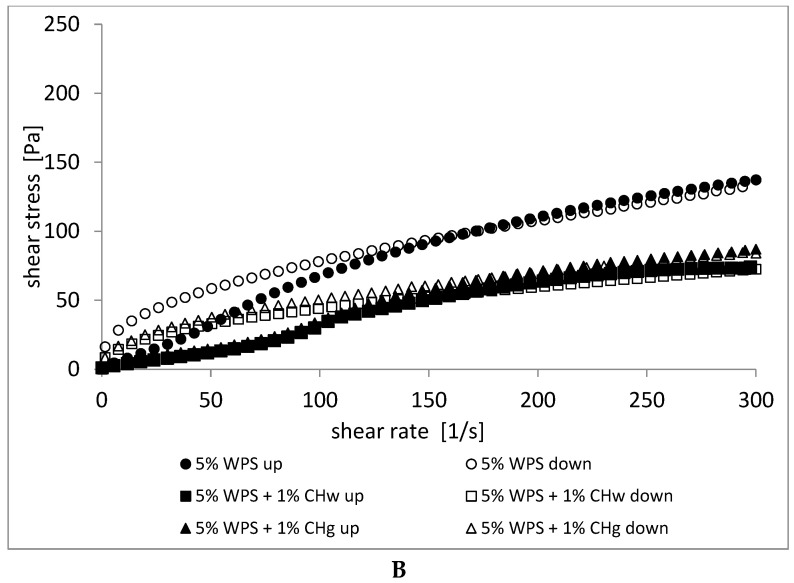
Flow curves of (**A**) 5% PS pastes and its mixtures with 1% CH (whole and ground) and (**B**) 5% WPS pastes and its mixtures with 1% CH (whole and ground). “Up” and “down” mean curves of a hysteresis loop in the range of shear rates 0–300 s^−1^ and 300–0 s^−1^, respectively.

Figure 2 shows the flow curves of natural (A) and waxy potato starch (B) pastes and their mixtures with the whole and ground chia seeds. The obtained flow curves were described by the Oswald-de Waele model for increasing curve of hysteresis (“up” curve 0–300 s^−1^) and decreasing (“down” curve 300–0 s^−1^) shear rates and are summarized in Table 3 and Table 4. The values of the consistency index (*K*) and flow behavior index (*n*) of the starches and their mixtures with chia seeds were calculated for upward and downward curves. The rheological model used was characterized by a very good fit to the experimental data (R^2^ > 0.998), showing that the model was adequate to determine the flow behavior of the starch pastes with chia seeds.

The highest shear stress was found in normal and waxy starch pastes without the addition of chia seeds at both increasing and decreasing shear rates. Moreover, the hysteresis loop for 5%PS was the greatest, which means that the sample is low rheologically stable [58,59]. After adding chia seeds, the shear stress values decreased significantly and areas of the loops as well (Figure 2A,B).

Results showed that the shear stress of starch-chia mixtures decreased distinctly compared to starch alone, which meant that the apparent viscosity also lowered. According to Campanella et al. (1995) [60], such behavior may be because as the shear rate increases, the polymers disintegrate (colloidal aggregation breaks down) and causes less resistance.

The coefficient of consistency (*K*) of the tested normal starch pastes showed a large differentiation with increasing (0–300 s^−1^) and decreasing (300–0 s^−1^) shear rates (Table 3). The values of the *K* coefficient at increasing shear rates amounted to 1.27 Pa∙s^n^ for the sample without the addition of chia seeds. The addition of chia increased the *K* coefficient to 1.31 Pa∙s^n^ in 5%PS + 1%CH_w_ and 7.27 Pa∙s^n^ in 5%PS + 1%CH_g_ samples. At decreasing shear rates, the *K* coefficient in normal potato starch paste, without the addition of chia seeds, was 12.52 Pa∙s^n^. After adding chia to starch, the coefficient pastes decreased to 4.05 Pa∙s^n^ for the sample with whole seeds and to 4.58 Pa∙s^n^ for the sample with ground chia seeds.

In the case of waxy starch pastes (5%WPS), significant changes in the *K* coefficient of 2.23 Pa∙s^n^ with increasing and 9.80 Pa∙s^n^ with decreasing shear rates were also observed. In both ranges, the addition of chia to waxy starch pastes resulted in a decrease in the consistency coefficient to 0.71 Pa∙s^n^ and 5.47 Pa∙s^n^ (5%WPS + 1%CH_w_) and 0.76 Pa∙s^n^ and 6.70 Pa∙s^n^ (5%WPS + 1%CH_g_), respectively (Table 4).

For all samples, the values of *n* were lower than 1 (Table 3 and Table 4), indicating the pseudoplastic properties of pastes [58,61], and the samples become thinner as the shear rate increases. In addition, a flow index lower than 0.6 is considered the limit for the good perception of food in the mouth [62].

Normal potato starch paste without the addition of chia (5% PS) at increasing shear rates gave values of the *n* coefficient equal to 0.93. The addition of whole seeds caused a decrease of this value to 0.76 and of ground seeds to 0.45. On the other hand, the values of the coefficient *n* at decreasing shear rates remained at the same level in the range of 0.52–0.54 for the examined pastes (Table 3), which indicates the pseudoplastic and shear thinning behavior of all mixtures.

The values of the coefficient *n* of waxy starch pastes at increasing shear rates were 0.72. The addition slightly increased the *n* value to 0.83 for the whole and 0.85 for the ground chia seeds. With decreasing shear rates, the *n* coefficient of waxy starch mixture with no additive was 0.50, and the addition of chia (whole and ground seeds) lowered this value to 0.45 (Table 4).

The pseudoplasticity was the result of an orientation effect. As the shear rate increased, the polymer molecules, which were long and randomly arranged strings, stretched and aligned in the direction of flow, resulting in less interaction between the adjacent polymer chains [63]. The higher the applied stress, the greater the ordering, lowering the apparent viscosity of samples [64]. The increase in pseudoplasticity of obtained mixtures involving chia seeds could have been caused, according to Goh et al. (2016) [19], by the presence of soft microgel particles derived from chia, which were very sensitive to shear and rapidly oriented in the direction of flow even at very low shear. Another reason, confirmed by studies of pasting characteristics (Figure 1), may have been the competition of soluble fractions in the mixture for water molecules and the limiting effect of fat on starch pasting. This could explain why the binary mixture showed lower viscosity than starch gel alone.

The measurement of the texture of starch gels is a test used to observe the retrogradation process in starch gels and their mixtures with various additions, e.g., with polysaccharide hydrocolloids [65]. Although there is not much information in the literature on the use of texture profile analysis to study the phenomenon of retrogradation of starch gels with and without the addition of hydrocolloids, this test can show changes in selected texture parameters (e.g., hardness) that are caused by short and long term retrogradation of starch gels [56,66,67,68,69,70]. Table 5 and Table 6 contain the hardness values of the normal and waxy starch gels and their gels with the addition of whole and ground chia seeds stored under refrigerated conditions of 4 °C for a period of 20 days, and the values are expressed in N.

In the case of normal potato starch (5% PS) on the day of starting the tests, the hardness of the gel without the addition of chia seeds was 0.60 N, with whole seeds at 0.96 N, and with the ground chia seeds at 0.77 N. After 1 day of storage, the samples without chia addition significantly increased their hardness to the value of 3.85 N. But the gels with the addition of chia seeds showed a lower increase in hardness. In the following days of storage (20 days), the hardness increased regularly. The highest hardness (4.72 N) was achieved by normal potato starch gel without the addition of chia. The remaining gels had a hardness of 2.10 N (5%PS + 1%CH_w_) and 2.06 N (5%PS + 1%CH_g_), respectively (Table 5). The hardness values for the stored samples were calculated as a ratio of gel hardness after 20 days of storing and the day of gel preparation (0 days) (Day 20/0). The largest difference in the hardness of gels was observed in the sample without chia seeds, i.e., normal potato starch (5%PS) 7.87, while in the starch-chia gels, the values were lower, i.e., 2.19 (5%PS + 1%CH_w_) and 2.67 (5%PS + 1%CH_g_) because in the normal starch, amylose forms tough gels [71]. The lower values of differences in hardness during storing indicate the stability of the samples in time, which included chia seeds [56]. A better effect was observed in the normal potato starch gel with the addition of whole than ground seeds.

As shown in Table 6, the waxy starch gel was characterized by much lower hardness than normal starch gel (Table 5) because amylopectin forms soft gels [71]. On the first day of the test, the hardness of the 5%WPS gel without the addition of seeds was 0.18 N. The addition of seeds did not cause statistically significant changes in the hardness of the starch gel, which was 0.17 N for the whole and 0.23 N for the ground chia seeds. After the first day, the hardness of the waxy gels increased and doubled. In the following days, the hardness gradually increased until it reached the highest value on the 20th day of storage. In the case of samples without chia addition (5%WPS), the final hardness was 1.58 N. The highest hardness value, 2.68 N, was found for waxy starch with the addition of ground chia seeds. The gel with whole chia seeds showed a similar value equal to 2.51 N.

In the waxy potato starch gels, the hardness values for stored samples that were calculated as a ratio of gel hardness after 20 days of storing and the day of gel preparation (0 days) had an opposite trend than was described in the potato starch gel. The largest difference in the hardness of gels was observed in samples with chia seeds—14.76 (5%WPS + 1%CH_w_) and 11.65 (5%WPS + 1%CH_g_), while in the waxy starch gel, the value was lower, 8.78 (5%WPS). The higher values of differences in hardness during storing indicate non-stable samples in time (5%WPS + 1%CH_w_ and 5%WPS + 1%CH_w_).

Starch gels, as reported in the literature, may undergo retrogradation when stored at low temperatures, which consists of the formation of bonds between starch molecules and leads to an increase in crystallinity, which causes the gel to be hard [65,71,72,73]. The amylose contained in starch plays an important role in this process and is a key parameter controlling susceptibility to retrogradation on cooling [74]. Waxy starches deprived of amylose show low retrogradability compared to normal starches and gives fewer rigidity gels [75,76]. As can be seen with the passage of storage time, the gels of normal and waxy starches and their mixtures, with the addition of whole and ground chia seeds, showed greater hardness. In the studies conducted by Gambuś and Gumul (2004) [72], the stored starch gels with a higher amylose content were characterized by a higher hardness and degree of retrogradation than gels consisting of amylopectin. Amylose contained in the starch undergoes retrogradation already in the initial stage of storage, while in the case of amylopectin, this process is much slower [77]. The data shows (Table 5 and Table 6) that the highest hardness during 20 days of storage was achieved by normal starch gels without the addition of chia seeds. The addition of whole and ground chia seeds to the starch gel caused a reduction in storage hardness. In the case of waxy starch gels, the hardness increased after adding chia seeds. This may be related to the changes that occur during the storage of chia seeds, particularly their fatty acids, as well as the presence of dietary fiber and other chia seed components.

## 3. Conclusions

The characteristic of gelatinization and flow properties of starch pastes and also textural properties of potato starch gels (normal and waxy) were influenced by chia seeds. The presence of chia in the blends had a higher impact on normal potato starch pastes properties than waxy potato starch suspension. The addition of chia seeds affected the viscosity of the mixture, especially the maximum viscosity, as a clear flattening of the peak was observed. The data indicate also that adding chia seeds strongly limits the gelatinization of starch granules. Moreover, all the studied pastes (normal and waxy starches) exhibited non-Newtonian, shear-thinning properties with a hysteresis loop. The bigger areas of the loops were observed in starch pastes without chia; thus, from a rheological point of view, the samples were less stable than the mixtures with chia seeds. Our results showed that the form in which the chia seeds were added to the starch suspensions, whole or ground, had an impact on gels during storage. Chia seeds can be used as an additive in starch-based food to improve the texture of starch gels and during storage, and they enrich carbohydrate products in fiber, fat, protein, and bioactive substances.

## 4. Materials and Methods

### 4.1. Materials

Normal potato starch (PS) (Superior Standard, Wielkopolskie Przedsiębiorstwo Przemysłu Ziemniaczanego S.A., Luboń, Poland) and waxy potato starch (WPS) (Eliane 100, AVEBE FOOD, Veendam, The Netherlands) were used. PS had the following parameters: 81.77% d.m.; 31% amylose; 110 mg% total phosphorus; 0.20%protein; 0.12% lipids. WPS contained 86.1% d.m., <1% amylose, 80 mg% total phosphorus, 0.22%protein and 0.11% lipids. Chia seeds (CH) were purchased from the local hypermarket in Rzeszów (Poland) and used in whole (CH_w_) and ground (CH_g_) forms. The producer of seeds was Casa Del Sur (Peru). CH had the following chemical composition per 100 g d.m. (analyzed according to the methods in chapter 3.2.1.): 29.86 g fat, 41.31 g carbohydrates, 18.03 g protein, 4.49 g ash, 36.24 g total dietary fiber (insoluble 31.76 g, soluble 8.27 g).

### 4.2. Methods

#### 4.2.1. Chemical Analyses of Chia Seeds

The chemical compositions of chia seeds were assessed according to the AOAC (2006) methods: total protein content used the Kjeldahl procedure using the Büchi B324 extraction system (Büchi, Apeldoorn, The Netherlands), with a nitrogen to protein conversion factor of 6.25. Dietary fiber (soluble, insoluble, and total dietary fiber) used the enzymatic gravimetric method, and fat content used the Soxhlet method (Büchi B811), while ash content used carbonization. The total carbohydrate content was calculated by subtracting the sum of moisture, protein, fat, and ash percentages from 100%.

#### 4.2.2. Pasting Characteristics

Measurements of pasting characteristics of normal and waxy potato starches (5% *w*/*w* d.m.) and mixtures of 5% (*w*/*w*) starches with chia seeds (CH) (1% *w*/*w* d.m.) were taken using a Viscograph-E Brabender (Brabender GmbH & Co. KG, Duisburg, Germany). The aqueous suspensions of potato starch blends were prepared at ambient temperature, and pasting behavior was measured in the viscograph for 83 min. Pasting characteristics of the PS and WPS suspensions, as well as mixtures of 5%PS + 1%CH and 5%WPS + 1%CH were run with the following parameters: (a) heating from 30 to 95 °C heated at 1.5 °C/min; (b) maintaining samples for 5 min at 95 °C; (c) cooling from 95 to 50 °C with 1.5 °C/min with constant agitation at 75 rpm. Measurements were run in duplicate.

#### 4.2.3. Flow Measurements

Sample preparation: The aqueous suspensions of 5% *w*/*w* plain starch were agitated (with constant stirring at 400 rpm) for 10 min at room temperature and a further 30 min at 95 °C. Obtained gels were distributed to the device’s measuring system. Gels of plain and waxy starch with chia seeds (whole and ground) were prepared with proportions: 5% (*w*/*w*) d.m. of starch and 1% (*w*/*w*) d.m. of chia seeds.

Flow curves were carried out using an RS6000 (Gebrueder Haake GmbH, Karlsruhe, Germany) rheometer in the controlled rate of shear (CR) mode with a CC26 Ti measuring system and a 1.9 gap. Measurements were taken at a constant temperature of 50 °C. The rate of shear was raised from 0 to 300 s^−1^ for 10 min and maintained at the maximum shear rate for 1 min. Then the shear rate was decreased from 300 to 0 s^−1^ over 10 min. The flow curves up (the rate of shear from 0 to 300 s^−1^ within 600 s) and down (the rate of shear from 300 to 0 s^−1^ within 600 s) were fitted to the Ostwald–de Waele (1) model [58].

(1) $$\tau =K\cdot \dot{\;\gamma^{n}}$$

where: *τ*—shear stress (Pa), *K*—consistency coefficient (Pa∙s^n^), $\dot{\gamma }$—shear rate (s^−1^) and *n*—flow behavior index (-).

#### 4.2.4. Textural Properties

The texture of the PS and WPS gels and their mixtures with chia seeds were characterized by a CT3 Texture Analyzer, Brookfield (Middleboro, MA, USA), equipped with the Texture Pro CT V1.2 Build 9 software. The penetration test was used. Fresh gels obtained in the Viscograph-E were transferred to the plastic cylinders in the amount of 30 mL. Texture measurements were performed for fresh gels (after 1 h of storing) and after 1 day, 2, 3, 7, 10, and 20 days of storing at 4 °C. The samples were compressed with a cylinder probe (ø 25.5 mm) at a 2 mm/s speed. The trigger load was set at 0.01 N, and the target value was 10 mm. Gel hardness was defined as maximal force applied (N). The reported results were the average values of at least three replications.

### 4.3. Statistics

The experimental data were calculated using Statistica v.13.3 (StatSoft, Inc., Tulsa, OK, USA). The analysis of variance was done using Duncan’s test at a confidence level of α = 0.05.

## Figures and Tables

**Table 1:** Pasting characteristics of 5% PS and PS with an addition of 1% of CH (whole and ground seeds).

Sample	T_0_ [°C]	η_max_ [B.U.]	η_min_ [B.U.]	η_95 °C_ [B.U.]	η_95 °C after 5 min_ [B.U.]	BD [B.U.]	η_50 °C_ [B.U.]
5%PS	64.0 ± 0.1 ^a^	1802.0 ± 11.3 ^a^	733.0 ± 7.1 ^b^	983.0 ± 18.4 ^a^	806.5 ± 10.6 ^bc^	1069.0 ± 18.4 ^a^	927.5 ± 7.8 ^b^
5%PS + 1%CH_w_	61.1 ± 0.1 ^c^	1047.0 ± 36.8 ^b^	816.5 ± 33.2 ^a^	1000.5 ± 36.1 ^a^	887.5 ± 40.3 ^a^	230.5 ± 3.5 ^b^	1037.0 ± 36.8 ^a^
5%PS + 1%CH_g_	62.1 ± 0.1 ^b^	748.0 ± 25.5 ^c^	768.0 ± 25.5 ^b^	805.5 ± 7.8 ^b^	825.0 ± 2.8 ^ab^	70.0 ± 17.0 ^c^	1039.0 ± 4.2 ^a^

The values marked in the columns with the same letters do not differ statistically significantly at α = 0.05. T_0_ (°C)—temperature at the beginning of pasting; η_max_. (B.U.)—maximum viscosity; T_ηmax_. (°C)—temperature at maximum viscosity; η_95 °C_ (B.U.)—viscosity at 95 °C; η_95 °C after 5 min_ (B.U.)—viscosity at 95 °C after 5 min holding; η_min._ (B.U.)—minimum viscosity; BD (B.U.)—breakdown; η_50 °C_ (B.U.)—viscosity after cooling to 50 °C; B.U.—Brabender Units.

**Table 2:** Pasting characteristics of 5% WPS and WPS with an addition of 1% of CH (whole and ground seeds).

Sample	T_0_ [°C]	η_max_ [B.U.]	η_min_ [B.U.]	η_95 °C_ [B.U.]	η_95 °C after 5 min_ [B.U.]	BD [B.U.]	η_50 °C_ [B.U.]
5%WPS	67.6 ± 0.1 ^a^	1386.5 ± 0.7 ^c^	436.0 ± 1.4 ^b^	575.5 ± 3.5 ^c^	483.0 ± 5.7 ^c^	950.5 ± 2.1 ^b^	483.0 ± 0.0 ^c^
5%WPS + 1%CH_w_	62.2 ± 0.6 ^c^	1576.0 ± 26.9 ^b^	452.0 ± 8.5 ^b^	645.0 ± 2.8 ^b^	517.5 ± 5.0 ^b^	1124 ± 18.4 ^a^	539.5 ± 6.4 ^b^
5%WPS + 1%CH_g_	64.1 ± 0.5 ^b^	1509.0 ± 5.7 ^a^	528.0 ± 2.1 ^a^	687.5 ± 3.5 ^a^	578.5 ± 2.1 ^a^	980.5 ± 7.8 ^b^	607.5 ± 3.5 ^a^

The values marked in the columns with the same letters do not differ statistically significantly at α = 0.05. T_0_ (°C)—temperature at the beginning of pasting; η_max._ (B.U.)—maximum viscosity; T_ηmax._ (°C)—temperature at maximum viscosity; η_95⁰C_ (B.U.)—viscosity at 95°C; η_95⁰C after 5 min_ (B.U.)—viscosity at 95°C after 5 min holding; η_min._ (B.U.)—minimum viscosity; BD (B.U.)—breakdown; η_50⁰C_ (B.U.)—viscosity after cooling to 50°C. B.U.—Brabender Units.

**Table 3:** Parameters of steady flow measurements (Ostwald–de Waele model) of 5% PS and its mixtures with 1% CH (whole and ground seeds).

Sample	*K* (Pa∙s^n^)		*n* (-)		R^2^	
	0–300 s^−1^	300–0 s^−1^	0–300 s^−1^	300–0 s^−1^	0–300 s^−1^	300–0 s^−1^
5%PS	1.27 ± 0.05 ^b^	12.52 ± 0.00 ^a^	0.93 ± 0.01 ^a^	0.52 ± 0.00 ^a^	1.00 ± 0.00	1.00 ± 0.00
5%PS + 1%CH_w_	1.31 ± 0.31 ^b^	4.05 ± 0.04 ^c^	0.76 ± 0.02 ^b^	0.54 ± 0.02 ^a^	1.00 ± 0.00	1.00 ± 0.00
5%PS + 1%CH_g_	7.27 ± 0.97 ^a^	4.58 ± 0.19 ^b^	0.45 ± 0.02 ^c^	0.52 ± 0.01 ^a^	1.00 ± 0.00	1.00 ± 0.00

The values marked in the columns with the same letters do not differ statistically significantly at α = 0.05.

**Table 4:** Parameters of steady flow measurements (Ostwald–de Waele model) of 5% WPS and its mixtures with 1% CH (whole and ground seeds).

Sample	*K* (Pa∙s^n^)		*n* (-)		R^2^	
	0–300 s^−1^	300–0 s^−1^	0–300 s^−1^	300–0 s^−1^	0–300 s^−1^	300–0 s^−1^
5%WPS	2.35 ± 0.28 ^a^	9.80 ± 0.20 ^a^	0.72 ± 0.02 ^b^	0.50 ± 0.05 ^a^	0.99 ± 0.00	1.00 ± 0.00
5%WPS + 1%CH_w_	0.71 ± 0.04 ^b^	5.47 ± 0.40 ^b^	0.83 ± 0.03 ^a^	0.45 ± 0.01 ^a^	0.98 ± 0.01	1.00 ± 0.00
5%WPS + 1%CH_g_	0.76 ± 0.03 ^b^	6.70 ± 0.31 ^b^	0.85 ± 0.01 ^a^	0.45 ± 0.01 ^a^	0.99 ± 0.01	1.00 ± 0.00

The values marked in the columns with the same letters do not differ statistically significantly at α = 0.05.

**Table 5:** Gel hardness (N) of 5% PS and its mixtures with 1% CH (whole and ground) measured as fresh gels and gels stored for 20 days at 4 °C.

Sample	Hardness [N]							
	Fresh Gel (0 Day)	After 1 Day of Storage	After 2 Days of Storage	After 3 Days of Storage	After 7 Days of Storage	After 10 Days of Storage	After 20 Days of Storage	Day 20/0
5%PS	0.60 ± 0.02 ^c^	3.85 ± 0.44 ^a^	3.86 ± 0.15 ^a^	3.83 ± 0.43 ^a^	3.63 ± 0.25 ^a^	4.31 ± 0.05 ^a^	4.72 ± 0.2 ^a^	7.87 ^a^
5%PS + 1%CH_w_	0.96 ± 0.04 ^a^	1.93 ± 0.06 ^b^	1.89 ± 0.03 ^b^	1.76 ± 0.48 ^b^	1.44 ± 0.03 ^b^	1.77 ± 0.06 ^b^	2.10 ± 0.03 ^b^	2.19 ^c^
5%PS + 1%CH_g_	0.77 ± 0.01 ^b^	1.22 ± 0.02 ^c^	1.18 ± 0.06 ^c^	1.16 ± 0.05 ^b^	1.10 ± 0.04 ^c^	1.16 ± 0.02 ^c^	2.06 ± 0.30 ^b^	2.67 ^b^

The values marked in the columns with the same letters do not differ statistically significantly at α = 0.05. Day 20/0—value increase expressed as the ratio of gel hardness after 20 days and 0 days (fresh gel) of measurement.

**Table 6:** Gel hardness (N) of 5% WPS and its mixtures with 1% CH (whole and ground) measured as fresh gels and gels stored for 20 days at 4 °C.

Sample	Hardness [N]							
	Fresh Gel (0 Day)	After 1 Day of Storage	After 2 Days of Storage	After 3 Days of Storage	After 7 Days of Storage	After 10 Days of Storage	After 20 Days of Storage	Day 20/0
5%WPS	0.18 ± 0.01 ^b^	0.35 ± 0.01 ^b^	0.53 ± 0.08 ^b^	0.64 ± 0.07 ^b^	0.94 ± 0.02 ^c^	1.34 ± 0.40 ^b^	1.58 ± 0.33 ^b^	8.78 ^c^
5%WPS + 1%CH_w_	0.17 ± 0.01 ^b^	0.36 ± 0.02 ^b^	0.60 ± 0.01 ^ab^	0.80 ± 0.02 ^a^	1.06 ± 0.05 ^b^	1.27 ± 0.06 ^b^	2.51 ± 0.04 ^a^	14.76 ^a^
5%WPS + 1%CH_g_	0.23 ± 0.01 ^a^	0.46 ± 0.02 ^a^	0.67 ± 0.01 ^a^	0.80 ± 0.02 ^a^	1.30 ± 0.05 ^a^	1.85 ± 0.13 ^a^	2.68 ± 0.07 ^a^	11.65 ^b^

The values marked in the columns with the same letters do not differ statistically significantly at α = 0.05. Day 20/0—value increase expressed as the ratio of gel hardness after 20 days and 0 day (fresh gel) of measurement.

## Data Availability

The data that support the findings of this study are available from the corresponding author upon reasonable request.
